# 

**Published:** 1880-07

**Authors:** 


Thad S. Up de Graff, M. D., Editor & Proprietor.

### CONTENTS FOR JULY, 1880.

**CONTRIBUTIONS.** PAGE.

Where Shall We Recreate and How?..........BEN. H. PRATT,........ 254

Building the Language of Eternity,..........AUSBURN TOWNER........ 257

Microscopic—II,..........S. O. GLASON, M. D........ 59

Exaggeration,..........J. B. CHANDLER........ 260

**MEDICAL ITEMS AND NEWS.**

Cotton-leaf Tea as Galactagogue—Painful Hæmorrhoids—Whooping Cough—Uticaria—Moles or Nævus—To Preserve the Strength of Magendie's Solution—Whooping Cough—Hydrophobia—Puerperal Eclampsia—Diptheria—Catgut Ligature—Gonorrhœa—Transfusion—Chorea—Night Sweats—Podophyllin—Diabetes—Fissured Nipples—Hydrate of Chloral, A Request—Bile in Urine—Coto Bark in Diarrhœa—Pulsatilla—Chest Diseases—Sims' Speculum—Cutaneous Affections—Alopecia and Psoriasis—Ringworm—Facial Neuralgia—Erectile Tumor—Puerperal Malarial Fever—Chronic Nervous Exhaustion—Sneezing—Diphtheria—Hypodermic Syringe Case—Hydrobromic Ether—Night-Sweating of Phthisis.......... 262 to 268

**HOUSEHOLD HINTS AND RECEIPES.**

Powder for Sweaty Feet—Protection of Iron Castings—Making Boots Waterproof—Liquid Glue—Tobacco Smoke for Gapes in Chickens—To Clean Celluloid Collars—Gum Euphorbium as a Coating for Submerged Iron—Remedy for Corns—Postage Stamp Mucilage—A Good Blacking—Good Paste, Very Cheap—Turner's Cement—Kerosene Oil Lamps—Iron Cement for Closing the Joints of Iron Pipes—Sugar from Sorghum—How to Medicate a Pig—Purifying Honey.......... 269 to 270

**OUR CORRESPONDENCE** Eleven Letters and Answers.......... 271 to 273

**EDITORIAL.**

SCHOOL HYGIENE—II.......... 274

RAIN AND RHEUMATISM.......... 274

A LITTLE POLITICS.......... 276

CAMP NOTES.......... 181

**CHIT-CHAT.**

Married—The Camping Number—Kept our Promise—The Drum Major—The Elmira Microscopical Society—Locomotives and Churches—Ausburn Towner—A Pin Cushion—Microscopical—About Coroners—The New School Building—A Nest—Valorous but Indiscreet—Fritz—Edison—Household Duties—The Fourth—A Mimic—A Sucker—"What' in a Name"—The City—A Feat in Balancing—A Quiet Seat.......... 277 to 280

